# Enhanced Humidity Sensitivity with Silicon Nanopillar Array by UV Light

**DOI:** 10.3390/s18020660

**Published:** 2018-02-23

**Authors:** Wei Li, Chao Ding, Yun Cai, Juyan Liu, Linlin Wang, Qingying Ren, Jie Xu

**Affiliations:** 1 State-Province Joint Engineering Laboratory for RF Integration and Micropackaging, College of Electronic and Optical Engineering & College of Microelectronics, Nanjing University of Posts and Telecommunications, Nanjing 210023, China; 1016020826@njupt.edu.cn (C.D.); 1017020831@njupt.edu.cn (Y.C.); 1216022745@njupt.edu.cn (J.L.); 1217022824@njupt.edu.cn (L.W.); Rqy@njupt.edu.cn (Q.R.); jiexu@njupt.edu.cn (J.X.); 2 State Key Laboratory of Millimeter Waves, Southeast University, Nanjing 210096, China

**Keywords:** silicon nanopillar, humidity sensing, UV light

## Abstract

The sensitivity of silicon nanopillar array for relative humidity (RH) with UV illumination was investigated in this work. The silicon nanopillar array was prepared by nanosphere lithography. Electrical measurements were performed on its sensing performance with and without UV irradiation. It was found that UV light improved the humidity sensitivity with different UV light wavelengths and power. The sensor response and recovery time were reduced. Furthermore, the turn-on threshold voltage and the operating voltage both decreased. These sensing characteristics can mainly be attributed to the electron-hole pairs generated by UV light. These electron-hole pairs promote the adsorption and desorption processes. The results indicate that silicon nanopillar array materials with UV irradiation might be competitive as novel sensing materials for fabricating humidity sensors with high performances.

## 1. Introduction

The humidity sensor is one of the most important sensors that has been used extensively in our daily life. Unlike other gas sensors, which are used to detect organic vapour gas and hazardous gas, a humidity sensor can monitor the environmental moisture for human comfort. It can also be used in automotive, medical, and food processing industries. So, humidity sensors have attracted much attention in recent years [1,2,3,4,5,6,7,8]. Many materials have been extensively studied. Metal oxide semiconductor films, such as SnO_2_ [9,10], WO_3_ [11,12], ZnO [13,14], and TiO_2_ [15,16] have been utilized in a variety of different roles. Georgieva et al. reported that as-deposited layers with R_Sn/Te_ ranging from 0.4 to 0.9 exhibited characteristics as room temperature humidity sensors with very high sensitivity, good selectivity, fast response, and short recovery period [9]. Mahjoub et al. demonstrated that a high sensitivity to the relative humidity was observed from the size-controlled ZnO quantum dots (QDs) embedded into a SiO_2_ matrix [14]. Buvailo found that a TiO_2_/LiCl-based nanostructured thin film for a humidity sensor had a fast response time of about 0.75 s [16]. Although these metal oxide semiconductor film humidity sensors obtained high sensitivity, a short response time, and good long-term stability, the development of Si-based material sensors is highly desirable, because of their ease in integrating with the already existing Si integrated circuit technology. 

Meanwhile, many methods, such as applying a high electric field across the sensor terminals [17], doping noble metals in the metal oxide [18], and illuminating the gas sensor with UV radiation [19], were used to further improve the sensing characteristics. Among these methods, it has been proved that UV irradiation is an efficient and inexpensive way to improve the sensing activity [20,21,22]. 

In previously published papers, it has been reported that an effective pathway for gas transportation could be built into a silicon nanopillar array (Si-NPA) sensor, which shows high humility sensitivity, a short response/recovery time, and a low operating voltage [23,24,25,26]. In this work, the UV irradiation approach mentioned just above was applied to improve the Si-NPA sensor humidity sensing activity. The electrical measurements were performed with and without UV irradiation at a relative humidity ranging from 30% to 90% at room temperature. The results showed the Si-NPA had a higher sensitivity and a faster response and recovery time with UV light. Furthermore, the turn-on threshold voltage decreased from 1 V to 0.5 V, and the operating voltage decreased from 3 V to 1 V. These sensing characteristics indicate that Si-NPA sensors with UV irradiation might be used in the future.

## 2. Experiment

**Figure 1:**
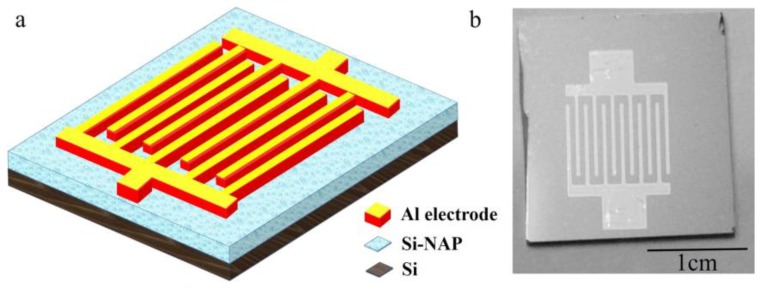
(**a**) The schematic diagram of the designed sensors. (**b**) A photo of the actual sample.

Si-NPA was prepared by nanosphere lithography. In the first step, a single layer of polystyrene (PS) spheres was covered on the P-Si substrate. The PS spheres with a diameter of 220 nm in the investigation were purchased from the Duke Scientific Corporation as 10% by wt. in solution with standard deviations between 5% and 7%. About 3 ~ 5 μL of solution that was diluted by mixing with an equal amount of ethanol was applied on the silicon substrate, which was kept in 10% dodecyl sodium sulfate solution for 24 h previously for a hydrophilic surface. Then, the substrate was slowly immersed into the deionized water at less than 45 degrees, and PS nanospheres formed an unordered monolayer on the water surface. Next, about 3 μL of 5% dodecyl sodium sulfate solution was added into the water in order to change the water surface tension, and an ordered monolayer was obtained. Last, the monolayer was then lifted off from the water surface to the silicon wafer. In the second step, the coated substrates with a pattern made of PS nanospheres were etched in the reaction ion etching (RIE) system (ME-3A, CAS) by using 20 sccm of O_2_ gas under a Radio Frequency (RF) power of 20W. In this RIE process, the size of the nanospheres was reduced. In the next step, the substrate was etched by using 40 sccm CF_4_ gas under a RF power of 40 W. Then, the large-area ordered Si nanopillar array can be obtained. Finally, the PS nanospheres were removed in tetrahydrofuran (THF). An aluminum comb-like electrode was prepared by electron-beam evaporation on the top of the Si-NPA. The process of EBV was performed at a 5 × 10^−4^ Pa vacuum, and the evaporation current was 25 mA. Figure 1 shows the schematic diagram of the designed sensor and a digital photo of the sensor. Humidity sensing measurements were performed in a quartz glass at room temperature (25 °C) under a UV light irradiation provided by a UV light-emitting diode.

## 3. Results and Discussion

**Figure 2:**
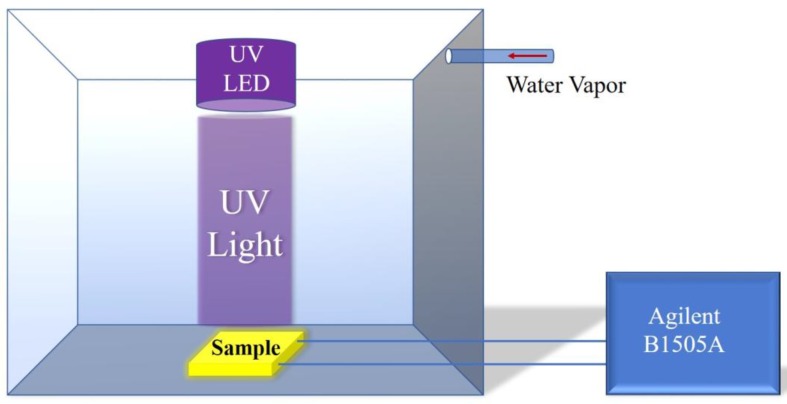
Measurement system.

Figure 2 shows the measurement system. The water vapor was taken into the glass chamber with a tube in order to change the relative humidity. The chamber is 30 cm × 30 cm × 30 cm. There is a reference humidity sensor to show the relative humidity (RH) in the chamber. At the beginning, the Si-NPA sensor was covered with a cover. When the relative humidity was kept to a certain degree, the cover was turned on/off to measure the humidity. The electrical resistance was monitored by using an Agilent (B1505A) electrometer (Agilent Technologies Inc., Santa Clara, CA, USA).

**Figure 3:**
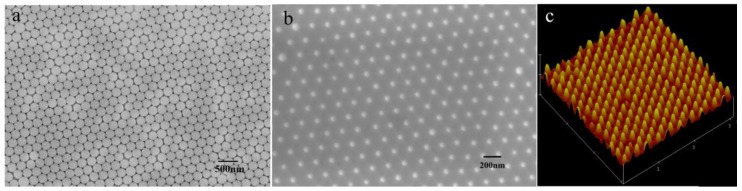
The morphology of the polystyrene (PS) monolayer and Si nanopillar array. (**a**) SEM image of the PS monolayer; (**b**) SEM image of the ordered Si nanopillar array; (**c**) and an atomic force microscope (AFM) image of the ordered Si nanopillar array.

Figure 3a,b shows a scanning electron microscope (SEM) image of the PS monolayer and the ordered nanopillar arrays. The SEM measurement was performed on a LEO1530VP (LEO Inc., Thornwood, NY, USA), and the AFM measurement was performed on a Nanoscope III (Digital Instrument Inc, Bresso, Italy.). Figure 3c is the oblique view of an atomic force microscope (AFM) image. As observed in Figure 3a, the typical morphology of a single layer ordered nanosphere array was obtained, and it is a hexagonally close packed (HCP) lattice structure with 220 nm in period. From Figure 3b, the periodic pattern is accurately transferred onto the Si substrate after RIE. The lattice constant is kept in 220 nm, which corresponds to the diameter of the PS spheres. As seen from the AFM image, the mean diameter of the Si pillars is about 50 nm, as measured on the top of the pillars, and the height of the pillar is about 120 nm. However, it is clearly observed that the side wall of the pillar is not vertical. The nanopillar is a cone type. 

**Figure 4:**
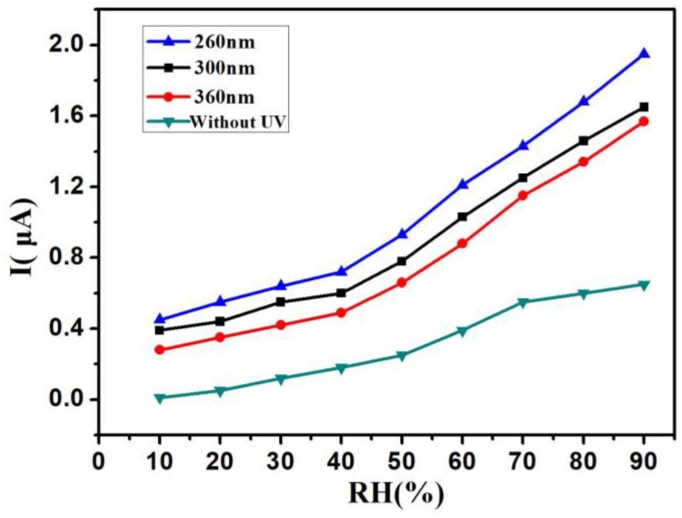
The current (I)-relative humidity (RH) curves at an applied voltage of 1 V with different UV wavelengths from 260 nm to 360 nm with the same power, 150 μW, and without UV irradiation.

**Figure 5:**
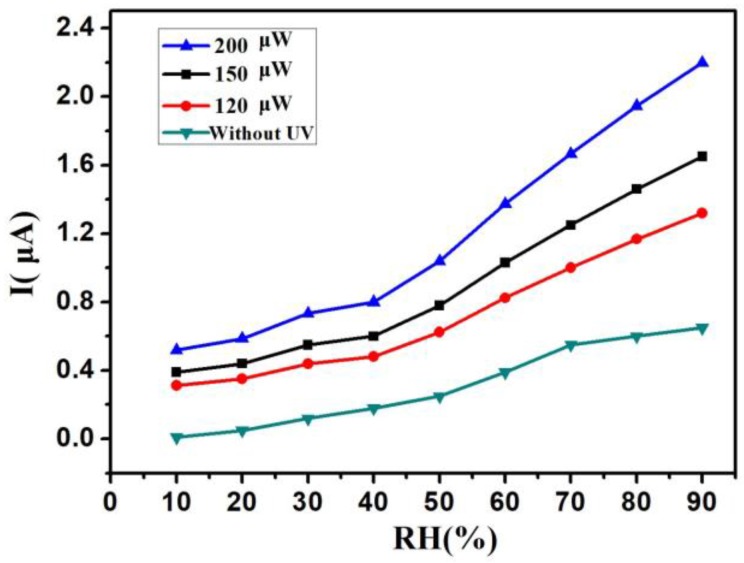
The I-RH curves at an applied voltage of 1 V with different UV power from 120 μW to 200 μW in the same wavelength, 300 nm, and without UV irradiation.

**Figure 6:**
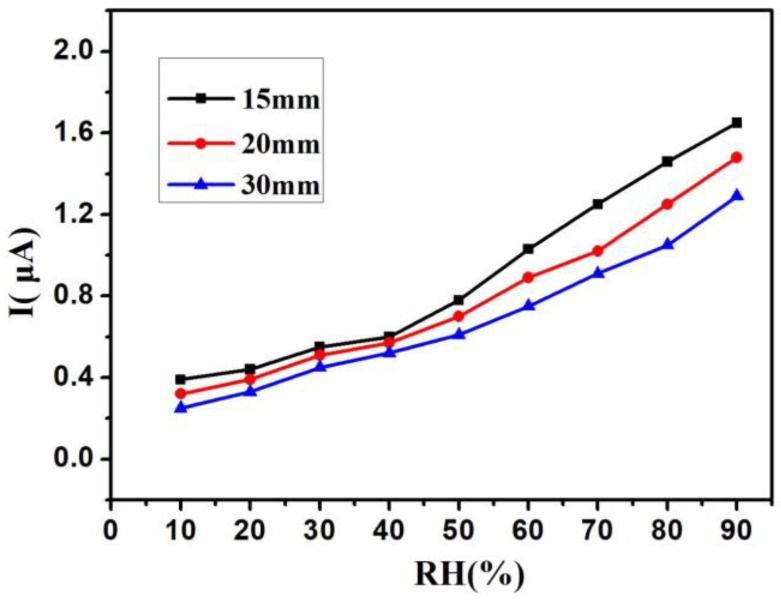
I-RH curves at an applied voltage of 1 V with different distances between the UV LED and the sample under UV LED (λ = 300 nm, P =150 μW).

The UV LEDs with different wavelengths and power were used in the work. Figure 4 shows the I–RH curves at an applied voltage of 1 V with different UV wavelengths—from 260 nm to 360 nm—in the same power of 150 μW, and without UV irradiation. Without UV irradiation, the current was just kept under 0.2 μA below 40% RH. The current was only 0.65 μA at 90% RH. With UV irradiation, the current was kept above 0.2 μA from 10% RH to 90% RH. These results indicate that UV irradiation improved the humidity sensitivity of the Si-NPA sensing materials. This UV-activated humidity sensing mechanism might most be attributed to the photogenerated electron-hole pairs induced by activated UV light. Without UV, the water vapor condensation happened through a capillary effect when the distance between Si pillars was only on a nanometer scale. The liquid water remained on the surface of the nanopillar, and there was limited surface diffusion of water molecules. As a result, the electrical resistivity of the Si nanopillar array sensors changed. With UV, the electrons and holes generated on the surface of the nanopillar changed the electrical resistivity of Si-NPA further, and made it easier for the composite system with the Si-NPA and liquid water to conduct electricity. While the I-RH curves had quite a small slope below 40% RH, the current increased rapidly above 40% RH, as shown in Figure 4. At 90% RH, the current rose from 1.5 μA to 2 μA when the UV LED wavelength decreased from 360 nm to 260 nm. The change of the current caused by the wavelength is not very evident. It can be reconciled with variations in the light adsorption: the longer wavelengths penetrated deeper into the Si-NPA sensing layer, and therefore induced more pronounced changes of its electrical resistivity, despite this light having lower energy than light at shorter wavelengths. Figure 5 shows the I–RH curves at an applied voltage of 1 V with different UV power from 120 μW to 200 μW in the same wavelength of 300 nm, and without UV irradiation. As the power increased, the current increased. Furthermore, the increase magnitude of the current was kept the same as the increase magnitude of the power. The change of the current caused by the power was due to the energy deposited from UV irradiation, and the electrical resistivity remained stable when the adsorption of the energy was saturated. From Figure 4 and Figure 5, the relative humidity could be detected under 10% RH by Si-NPA humidity sensing array with UV irradiation. Figure 6 shows the I–RH curves at an applied voltage of 1 V, with a different distance between the UV LED and the sample under UV light (λ = 300 nm, P = 150 μW). It was found that the change in the magnitude of the current was biggest at a 15-mm distance. It is attributed to the presence of more electron-hole pairs induced by UV light, which increased with the shorter distance. 

**Figure 7:**
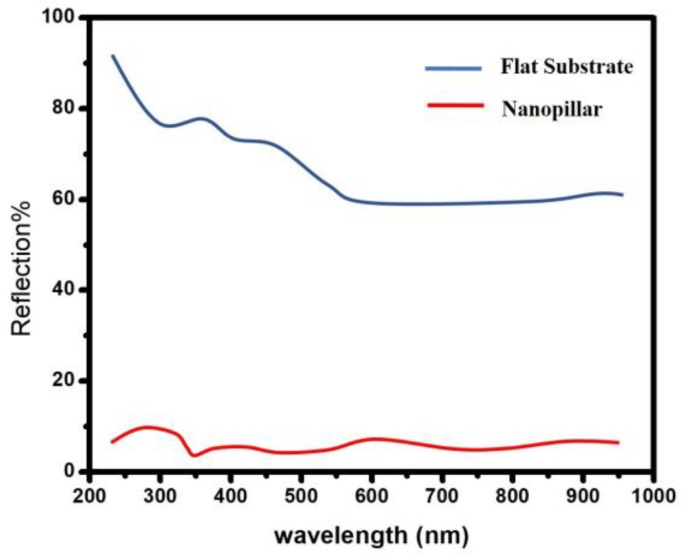
The reflectance spectrum for flat and Si nanopillar substrates.

Figure 7 shows the reflectance spectrum for flat and Si nanopillar substrates. The reflection spectra were measured with a Shimadzu UV-3600 spectrophotometer. It is shown that the reflectivity from the front surface of a flat Si substrate is above 50% in the whole measurement range. It is also interesting to find that the light reflection is obviously suppressed for the Si nanopillar samples. It is clear that the reflectivity is <10% in the UV wavelength range (from 200 nm to 400 nm), which means that more UV light was trapped in this nanostructure, and the photogenerated electron-hole pairs induced by UV light were substantially increased. This contributed to an improvement in the electrical resistivity.

**Figure 8:**
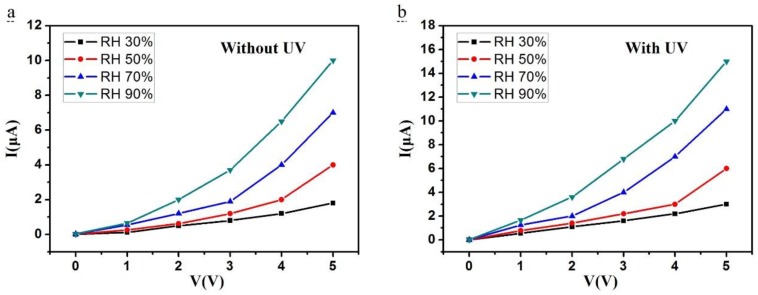
I-V curves measured from 30% RH to 90% RH. (**a**) Without UV irradiation; (**b**) With UV irradiation.

**Figure 9:**
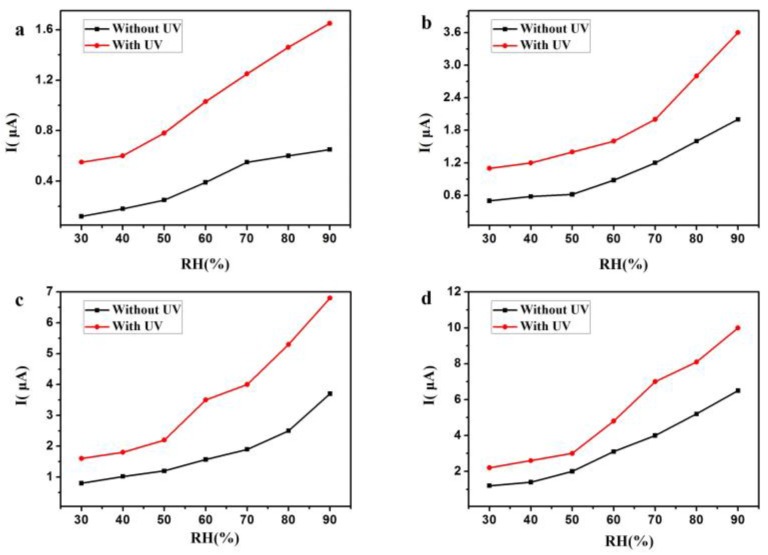
The humidity sensitivity from 30% RH to 90% RH at applied voltages from 1 V (**a**), 2 V (**b**), 3 V (**c**) and 4 V (**d**), with and without UV irradiation.

Figure 8a,b plots the current sensitivity of the Si nanopillar sensor versus the voltage at different RH (from 30–90%) with and without UV irradiation (LED, λ = 300 nm, P = 150 μW). As can be seen in this figure, the turn-on threshold voltage for this Si-NPA sensor was 1 V in Figure 8a, and 0.5 V in Figure 8b. While the I–V curves had quite a small slope below the applied voltage of 3 V, the current increased rapidly above an applied voltage of 3 V, as shown in Figure 8a. This voltage, 3 V, is defined as the operating voltage. However, the voltage was reduced to 1 V in Figure 8b. With UV irradiation, the turn-on threshold and the operating voltage were both reduced. Figure 9a–d shows the humidity sensitivity from 30% RH to 90% RH at applied voltages of 1 V, 2 V, 3 V, and 4 V, with and without UV irradiation. As observed, the current increased at any applied voltage with UV irradiation compared to that without UV irradiation. 

**Figure 10:**
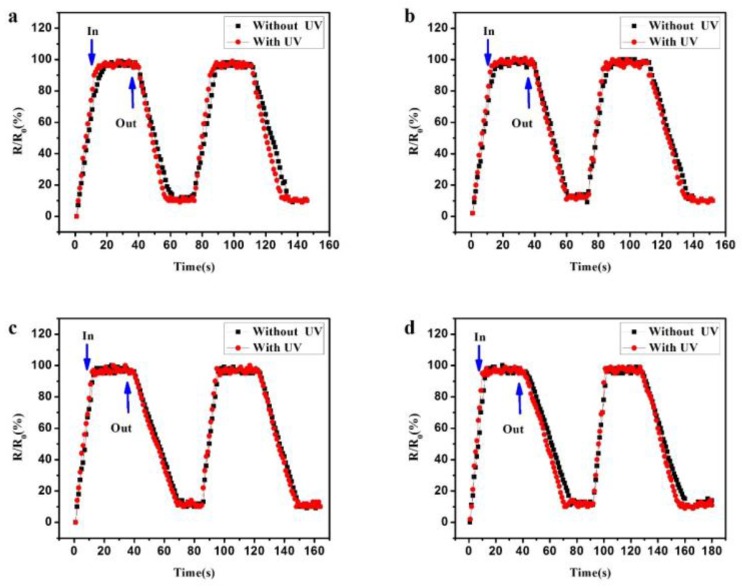
The response and recovery time at an applied voltage of 1 V, with and without UV irradiation: (**a**) 30%, (**b**) 50%, (**c**) 70%, and (**d**) 90%.

The response and recovery time was studied. Figure 10a–d shows the response and recovery time from 30% RH to 90% at an applied voltage of 1 V, with and without UV irradiation (LED, λ = 300 nm, P = 150 μW). R is the instantaneous resistance of this Si nanopillar, and R_0_ is the final/initial values. The response time is defined as the time spent from R/R_0_ = 10% to 100% when the sample was taken in the quartz glass. In contrast, the recovery time is defined as the time spent from R/R_0_ = 100% to 10% when the sample was removed from the quartz glass. Table 1 shows the detailed results of the response and recovery time. It was found that the response time was reduced, and the recovery time was increased as the RH increased. It is well known that water vapor remains in the gas phase at low relative humidity, and it will condense at high relative humidity. At high RH, the more water vapour was absorbed; as a result, it spent less time absorbing, and more time disrobing, these water molecules. Moreover, the response time and the recovery time were both reduced with UV light irradiation compared to without UV irradiation.

**Figure 11:**
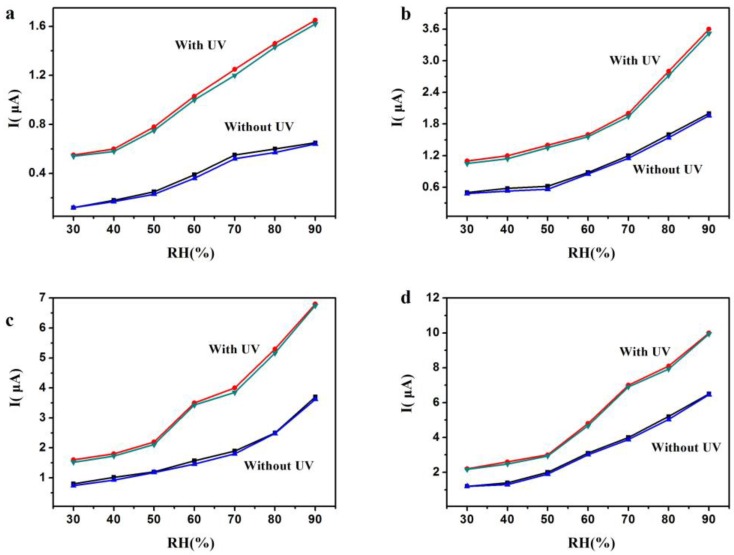
The humidity hysteresis measured for the sensors: (**a**) 1 V, (**b**) 2 V, (**c**) 3 V, and (**d**) 4 V.

The hysteresis was studied in the work, which refers to an important indicator of the performance of the humidity sensor. Figure 11a–d shows the current dependence on cyclic humidity changes at applied voltages of 1 V, 2 V, 3 V, and 4 V, with and without UV irradiation (LED, λ = 300 nm, P = 150 μW). Here, the upper curve represents the RH decreasing process, and the under curve stands for the RH increasing process. As seen in Figure 11, the two curves are very close for any sample, with or without UV light irradiation. It is indicated that the hysteresis for Si-NPA is small. The maximum humidity hysteresis is about ~2%, which occurred at 70% RH with an applied voltage of 3 V. Such a small hysteresis for the Si-NPA sensor should be due to its rapid response and recovery rates, and meet requirements for high-performance humidity sensors.

**Figure 12:**
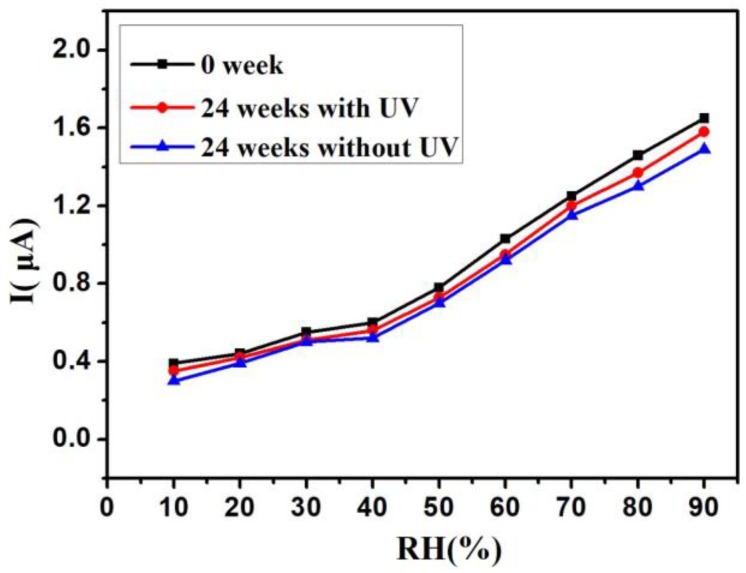
The humidity sensor stability after the sensors were exposed to air for 24 weeks with UV and without UV.

The stability is an important indicator of the performance of the humidity sensor, and the study of stability was performed after the sensors were exposed to air for 24 weeks. Figure 12 shows the humidity sensor stability. Compared with the samples exposed for 0 weeks, the curves measured after 24 weeks of storage both changed with UV irradiation (LED, λ = 300 nm, P = 150 μW) and without UV irradiation. For the sample with UV irradiation, the current drifts were about 5%, and the larger current drifts (about 12%) occurred in the sample without UV irradiation. It is considered that the active surface of Si-NPA could easily be oxidised at room temperature. The electrons and holes photogenerated by UV light can increase the conductive carriers. So, the stability of the humidity sensor with UV irradiation is superior to the sensor without UV irradiation.

## 4. Conclusions

In conclusion, the Si nanopillar array as a gas sensing material was fabricated by nanosphere lithography. The measurement of the room temperature humidity sensitivity of the Si nanopillar sensor without and with UV light irradiation was performed. The results show that the turn-on threshold voltage decreased from 1 V to 0.5 V, and the operating voltage decreased from 3 V to 1 V. Furthermore, UV light improved the sensitivity with different UV light wavelengths, power, and distance. The relative humidity could be detected under 10% RH, and the sensor response and recovery time were reduced. The hysteresis for Si-NPA with UV irradiation is small. The humidity sensor stability with UV irradiation is better. These sensing characteristics indicate that the Si-NPA with UV irradiation might be a promising candidate for fabricating high-performance humidity sensors.

## Figures and Tables

**Table 1:** The response and recovery time at an applied voltage of 1 V, with and without UV irradiation.

RH		30%	50%	70%	90%
Without UV	Response time	17 s	15 s	14 s	12 s
	Recovery time	23 s	26 s	30 s	35 s
With UV	Response time	15 s	13 s	11 s	10 s
	Recovery time	20 s	23 s	27 s	31 s
